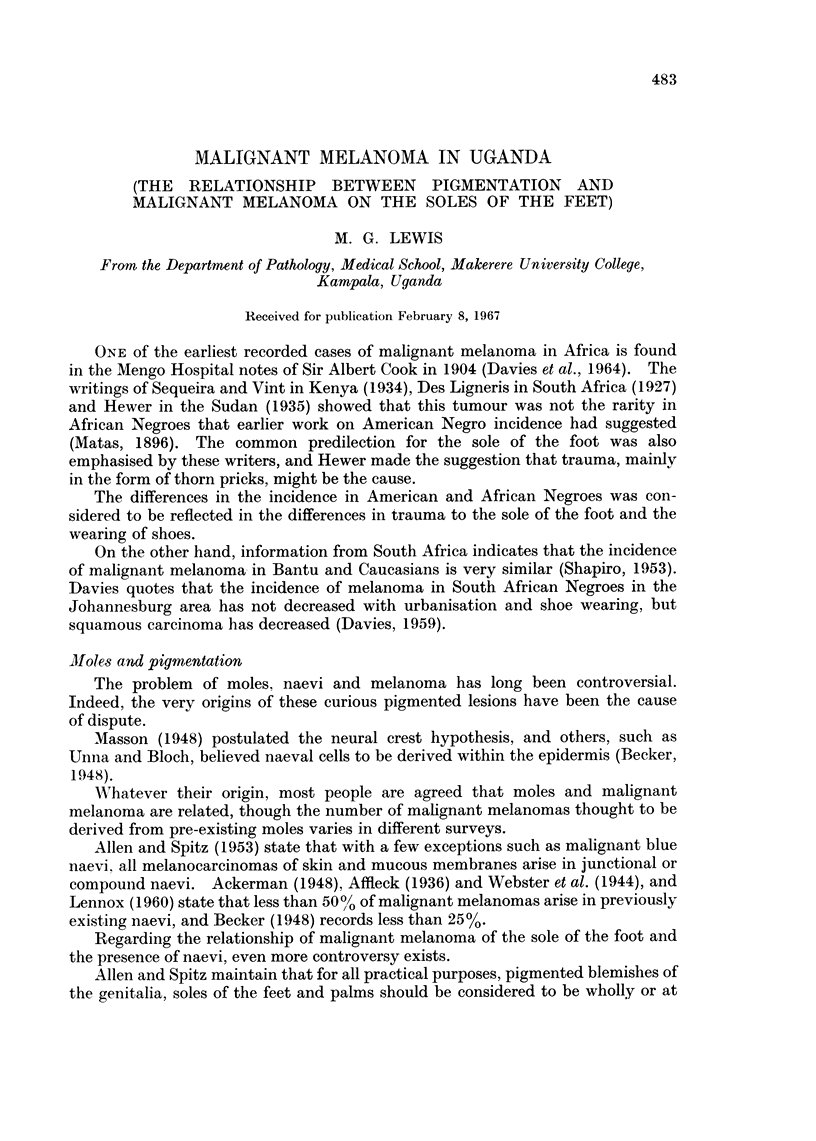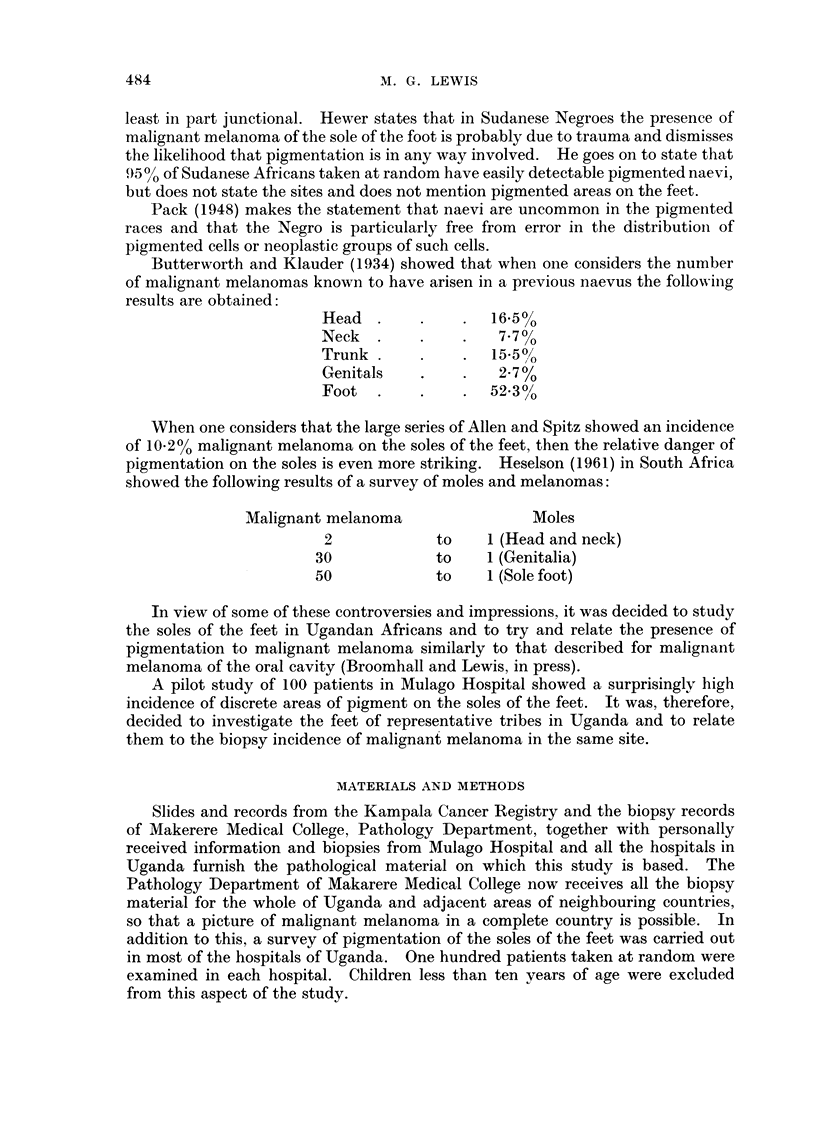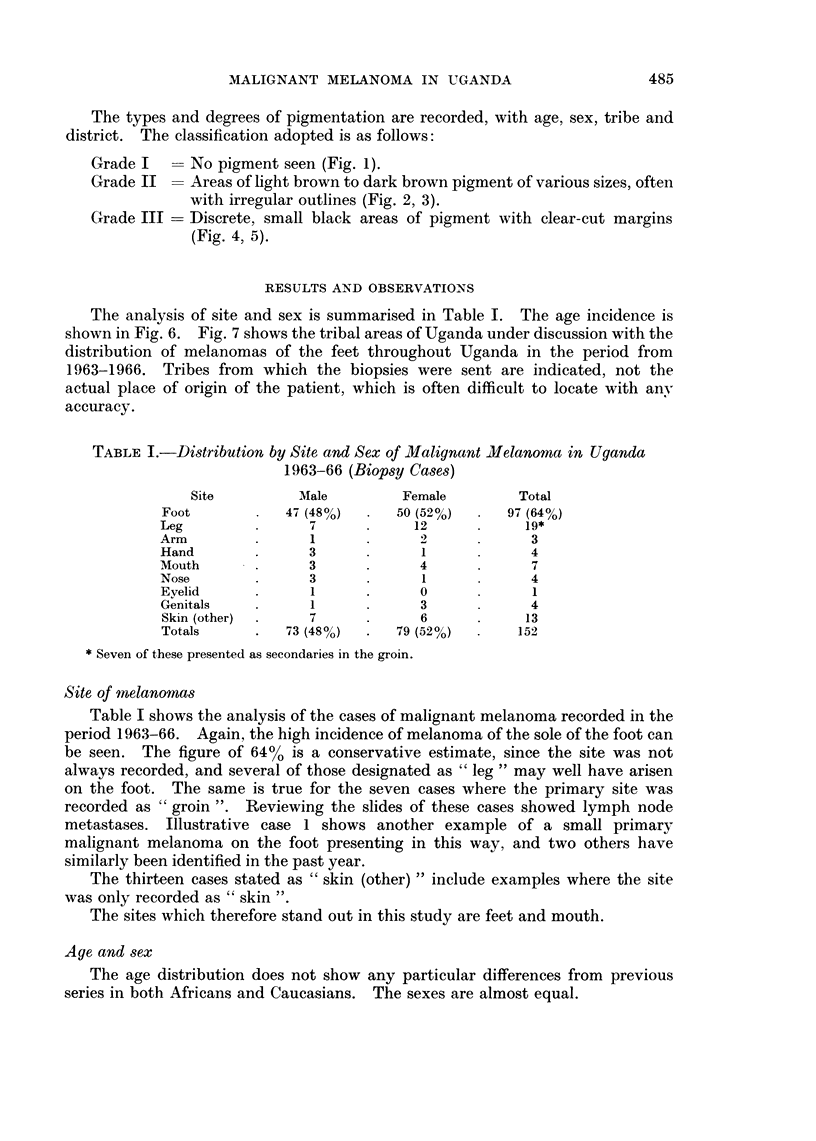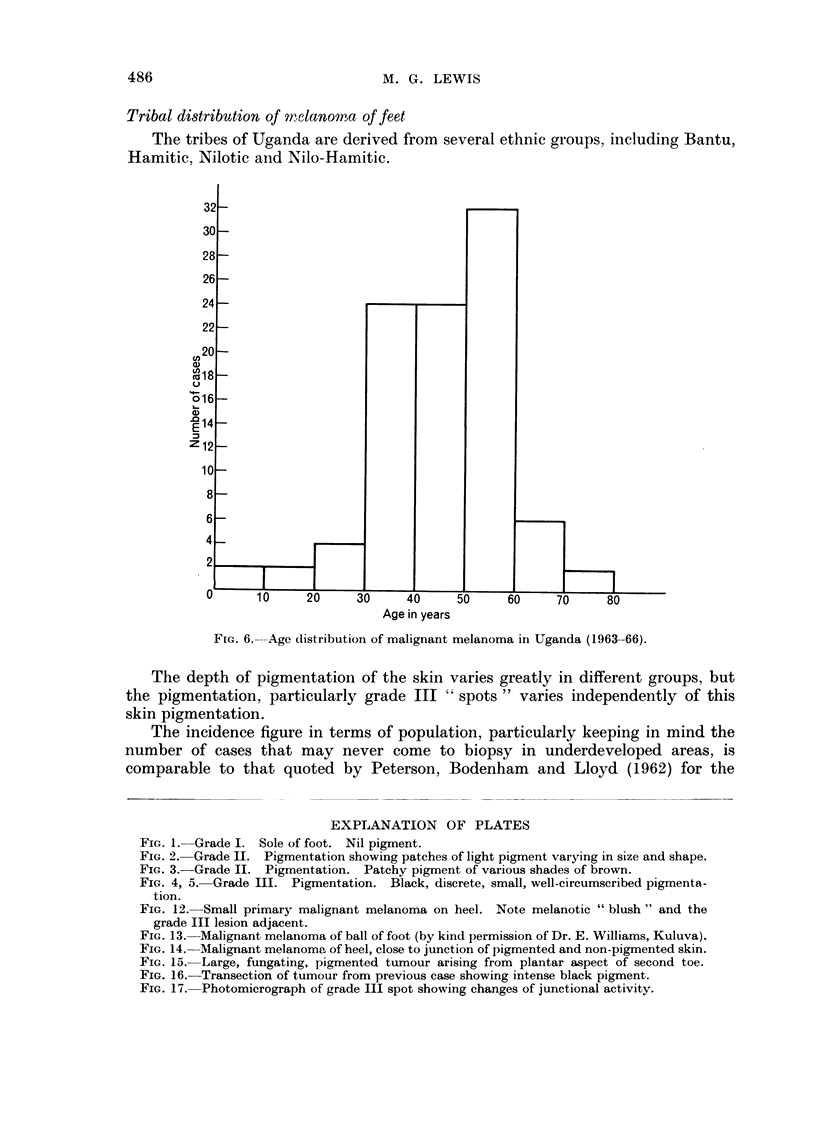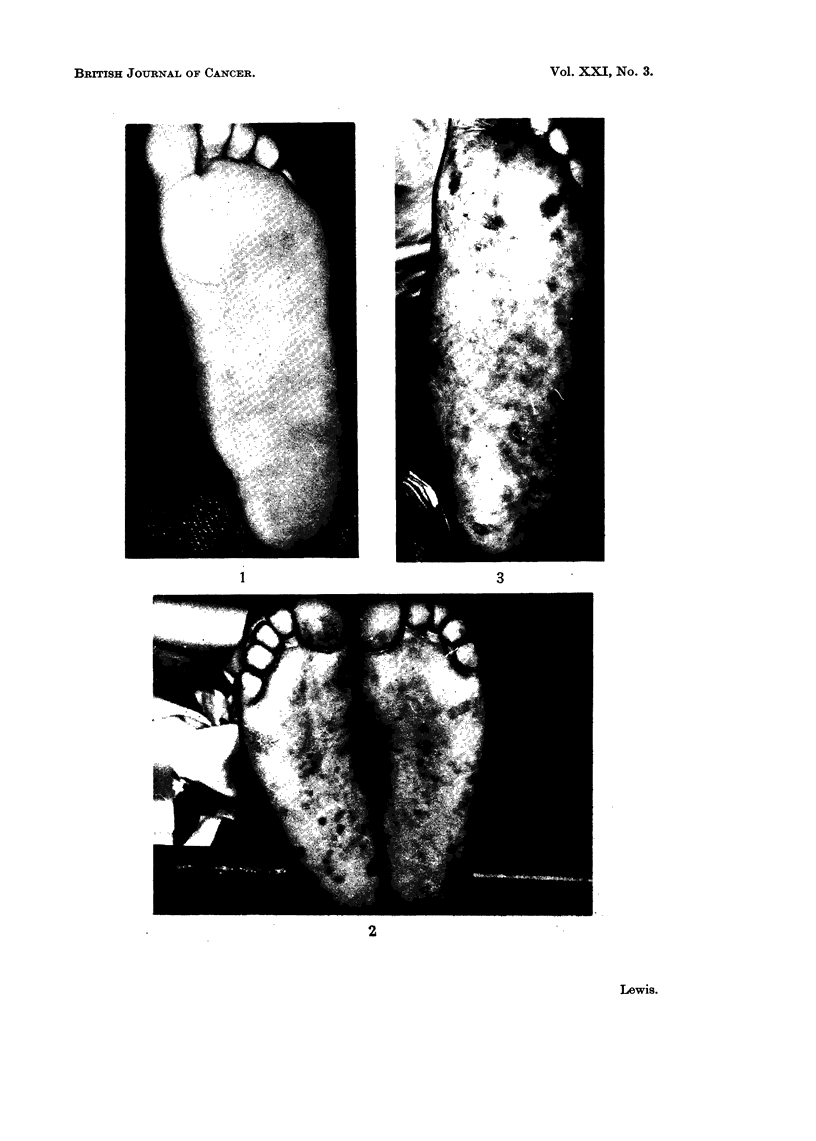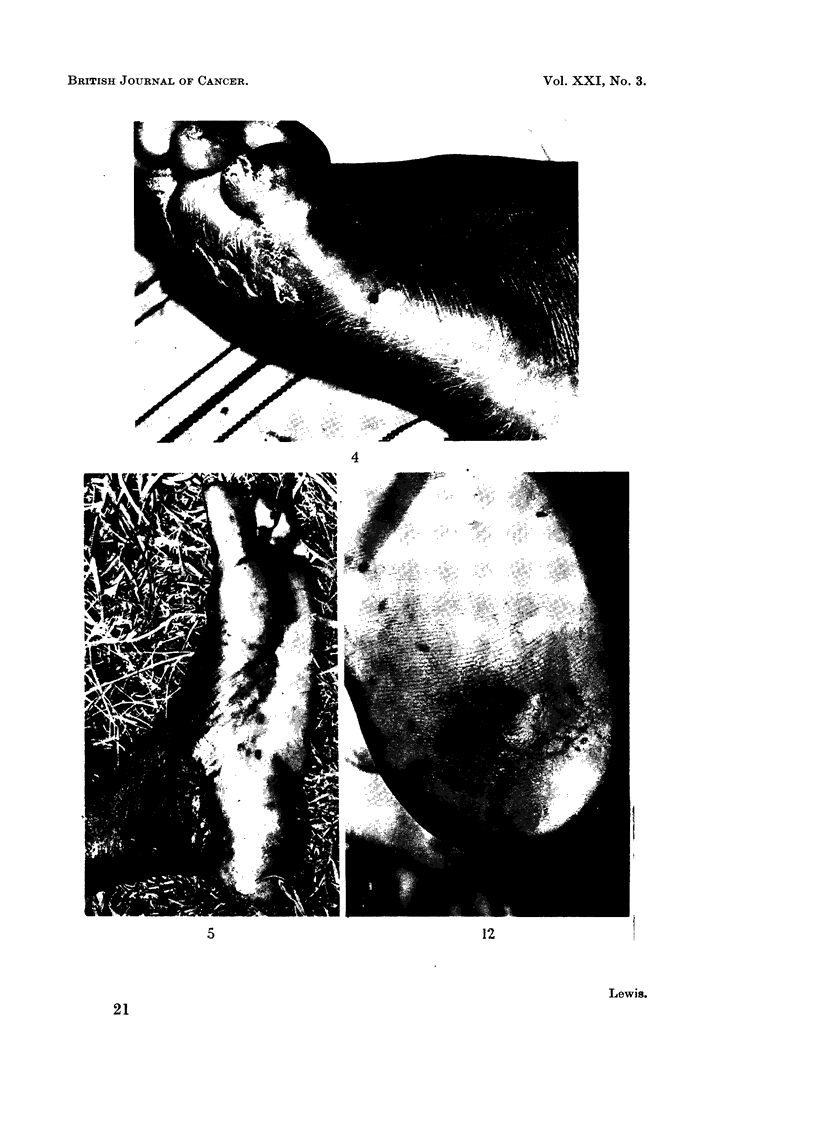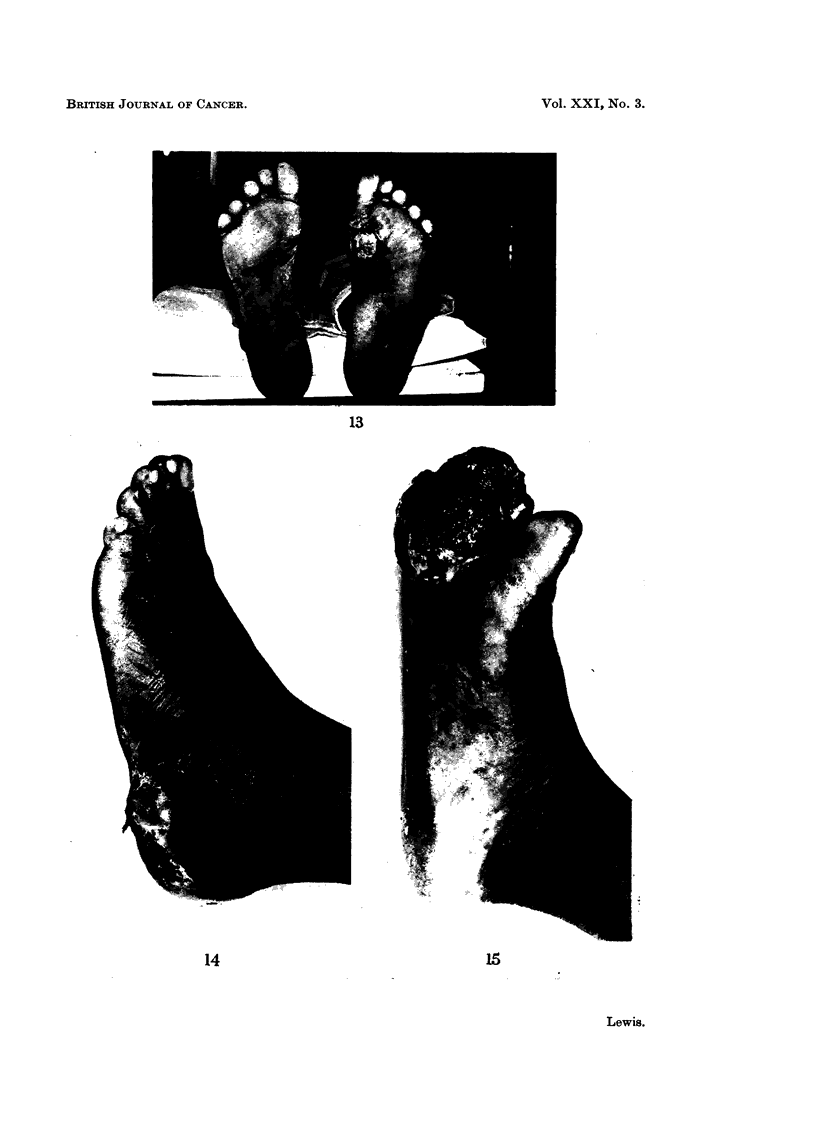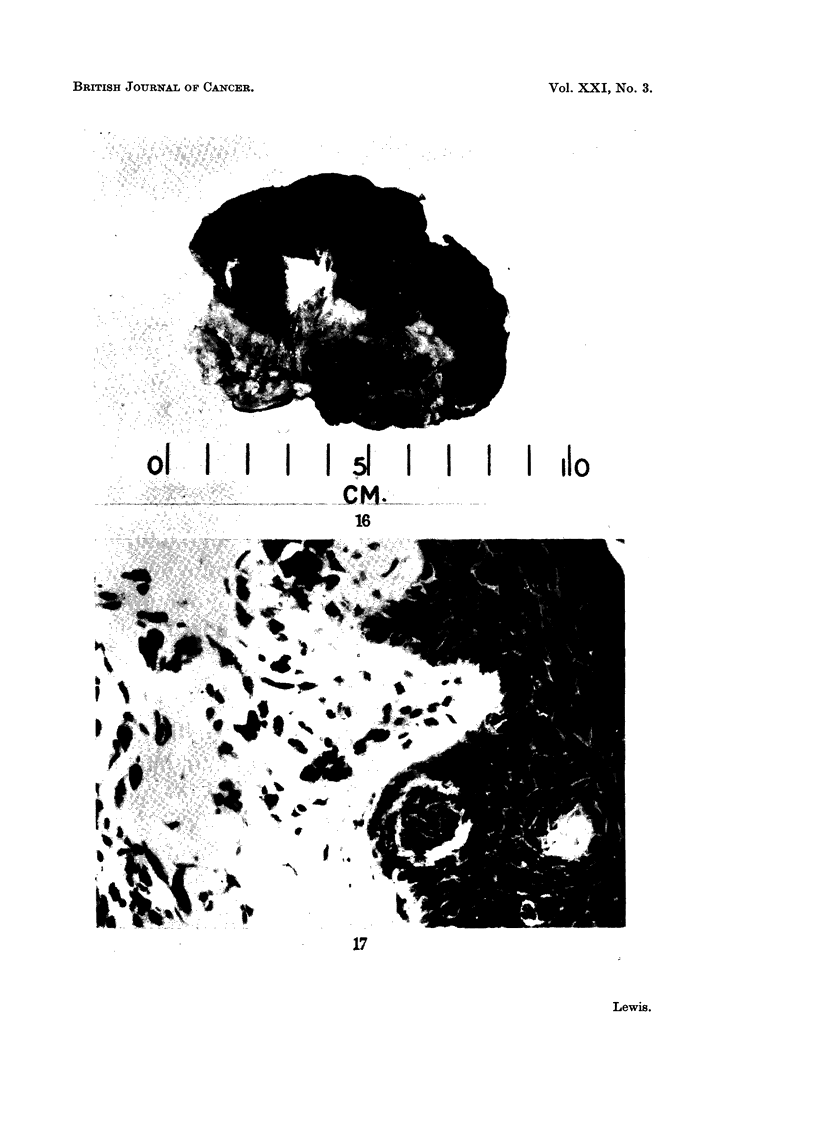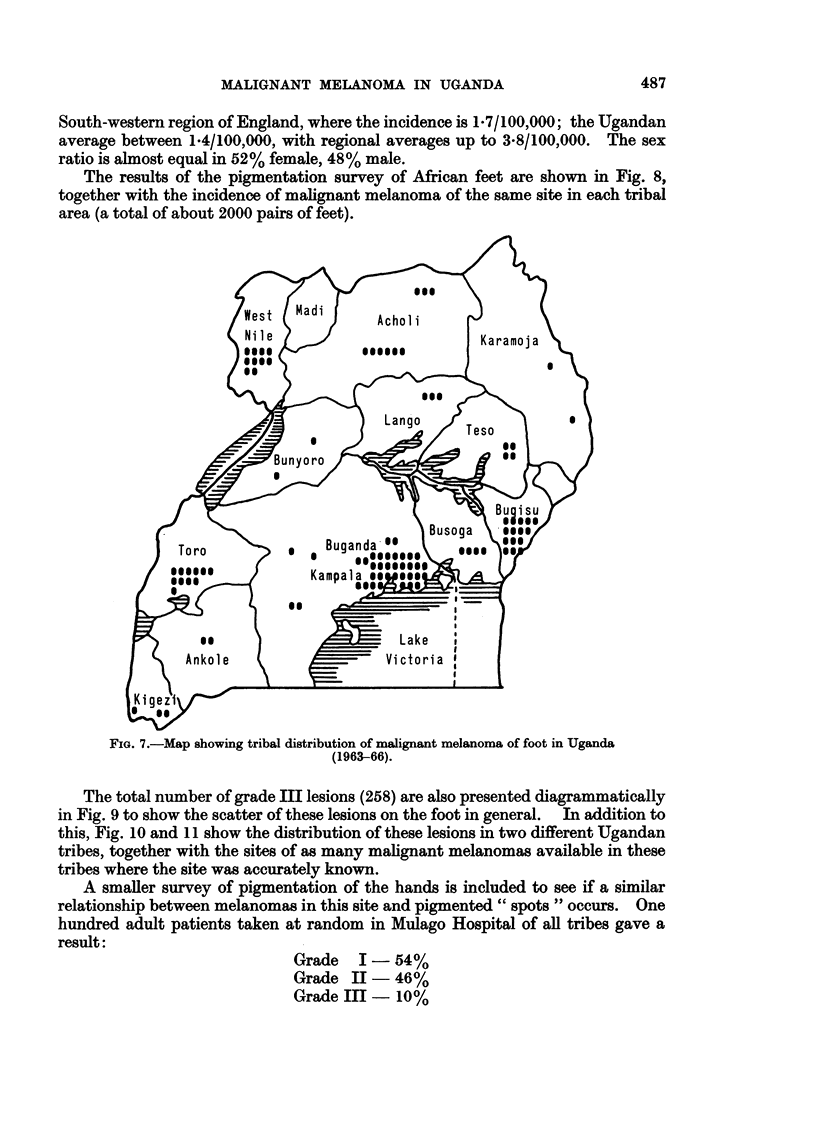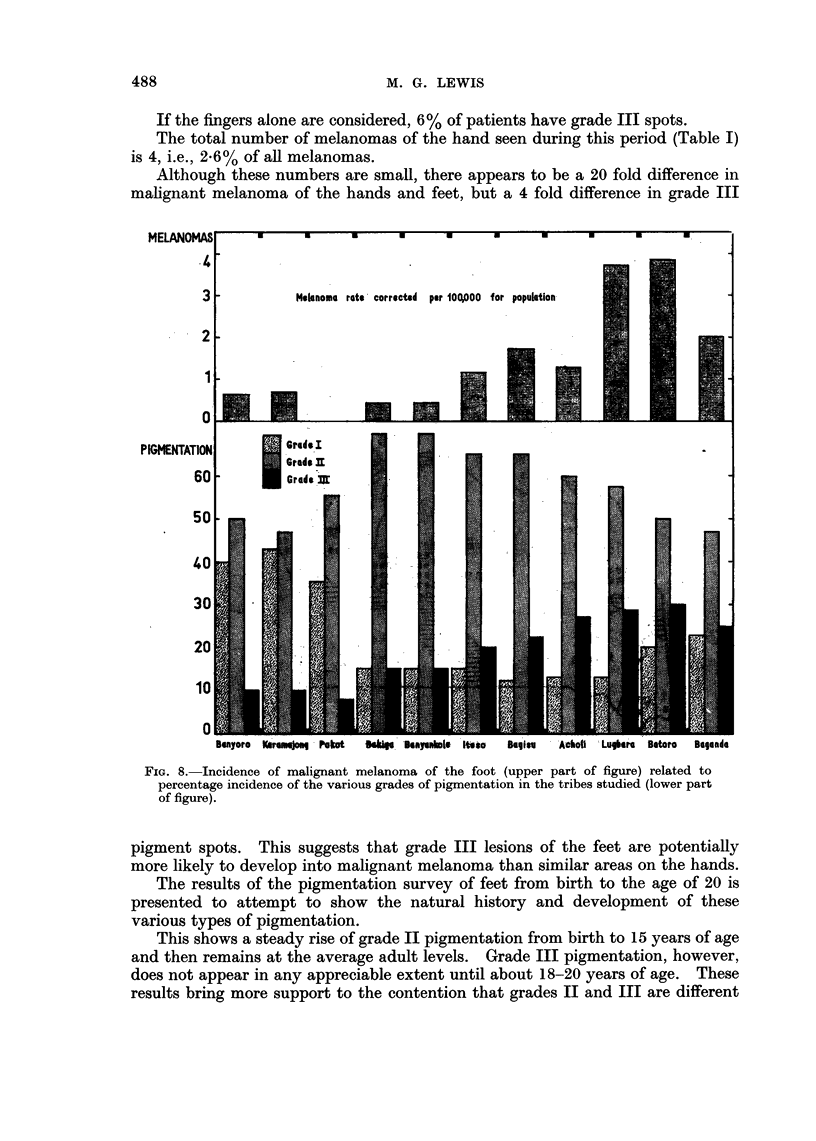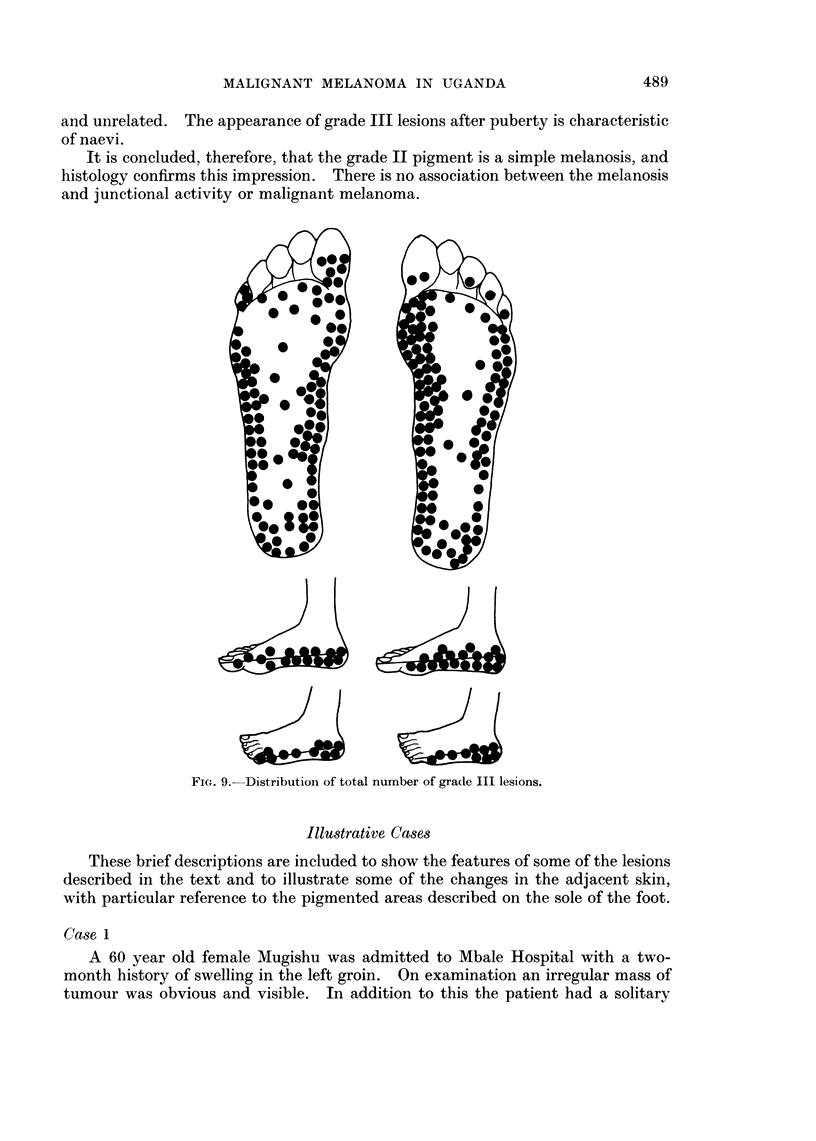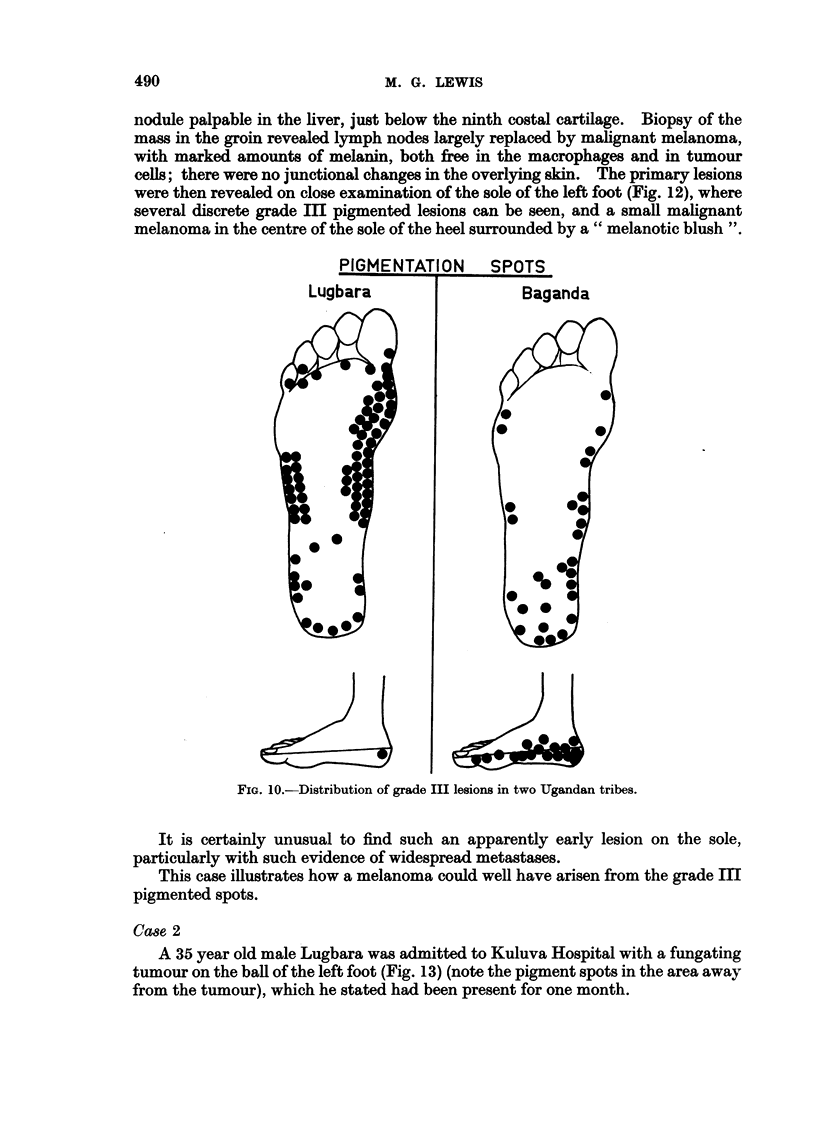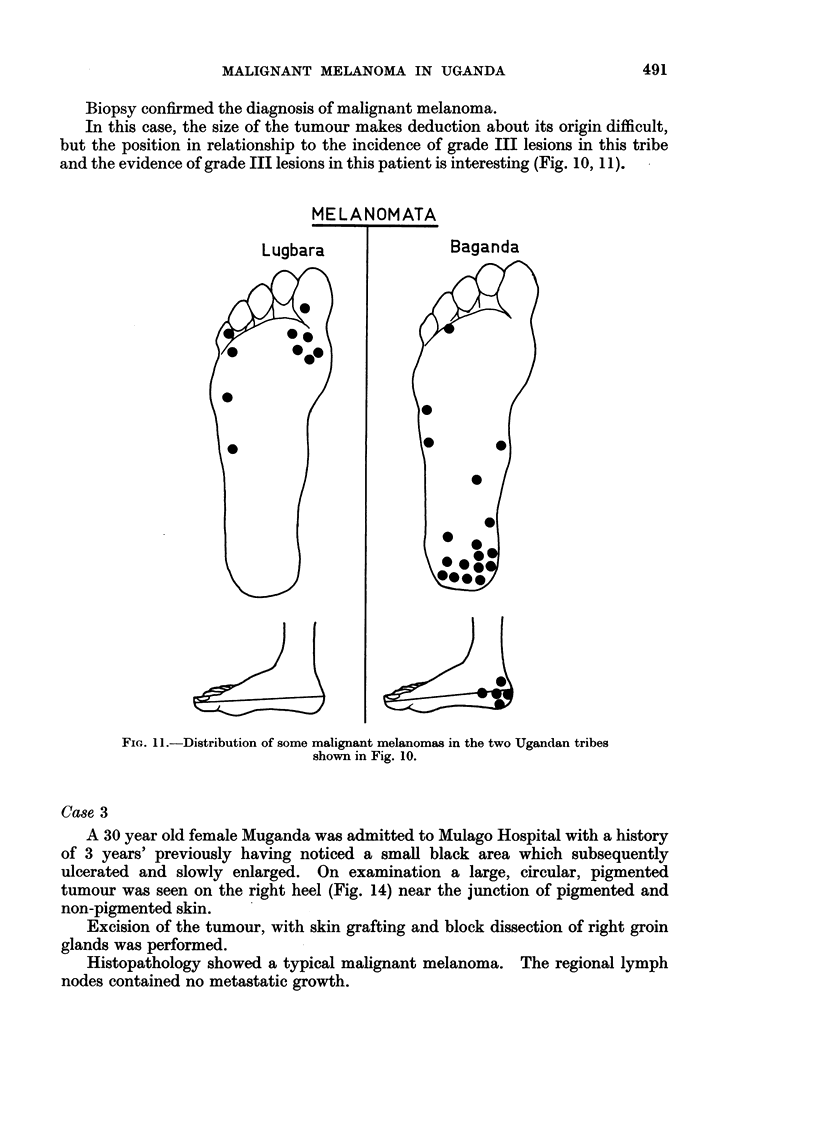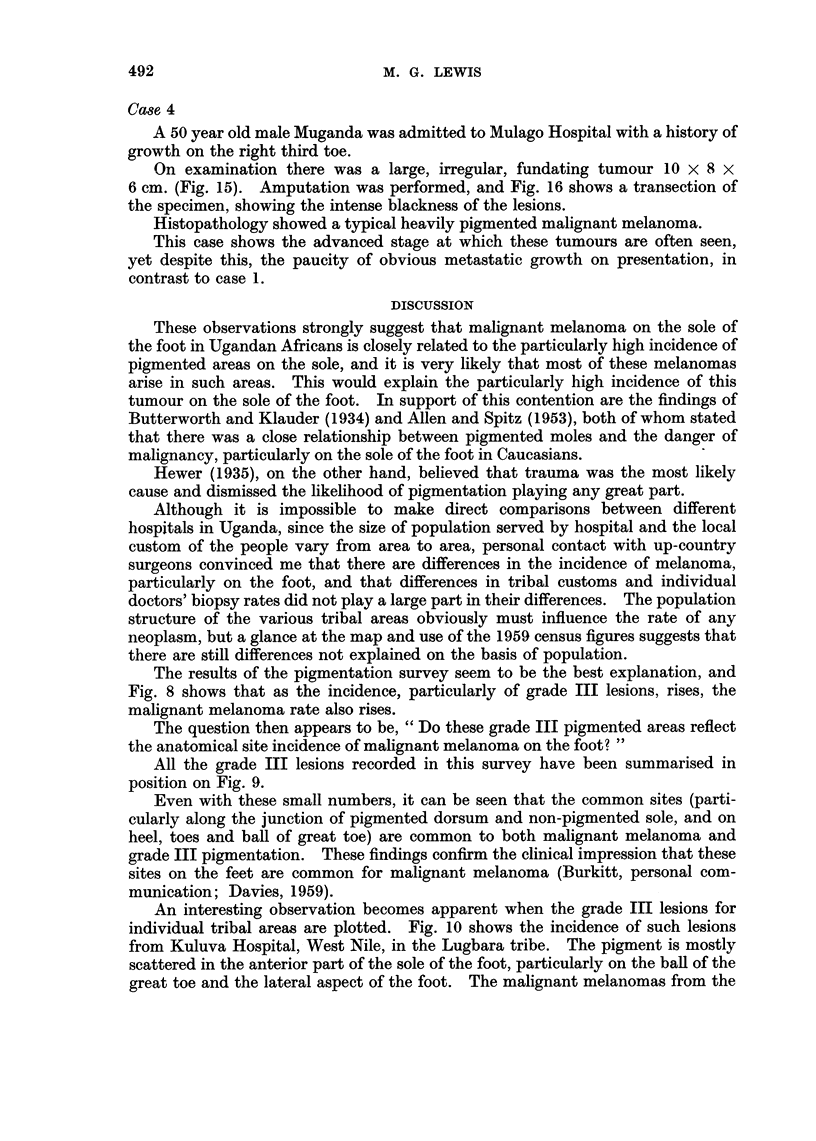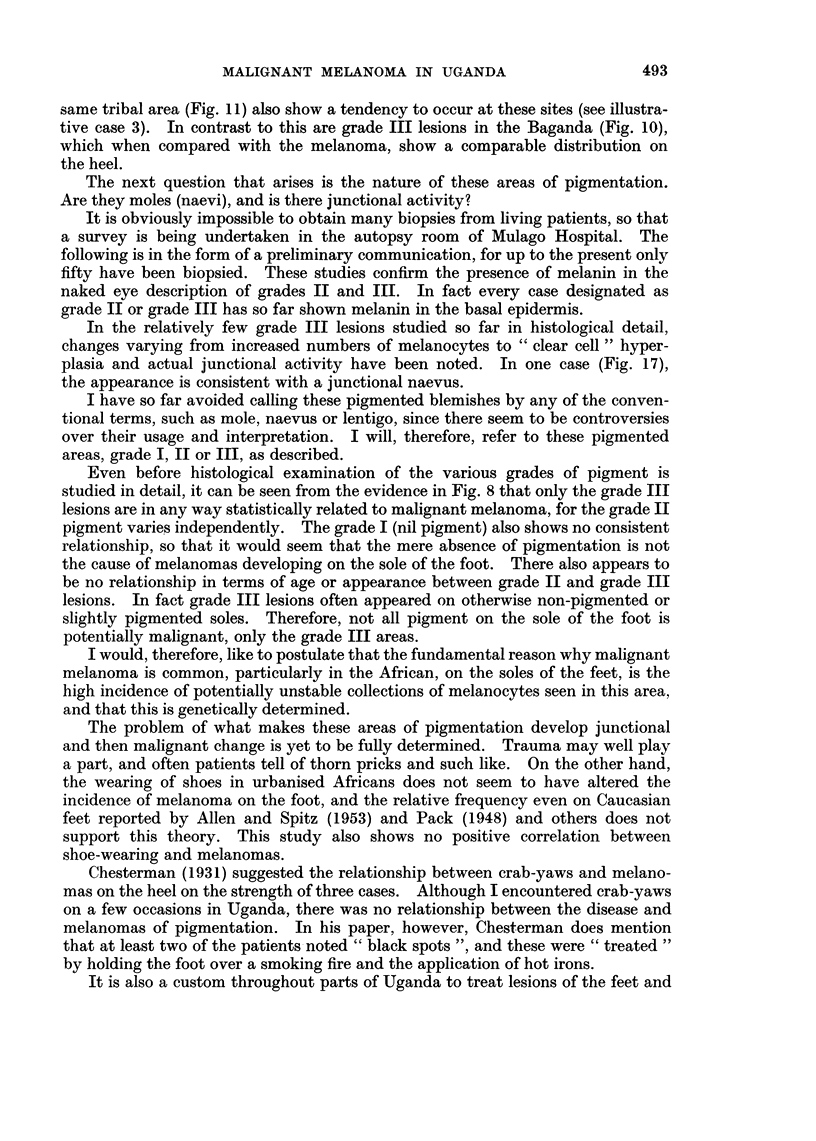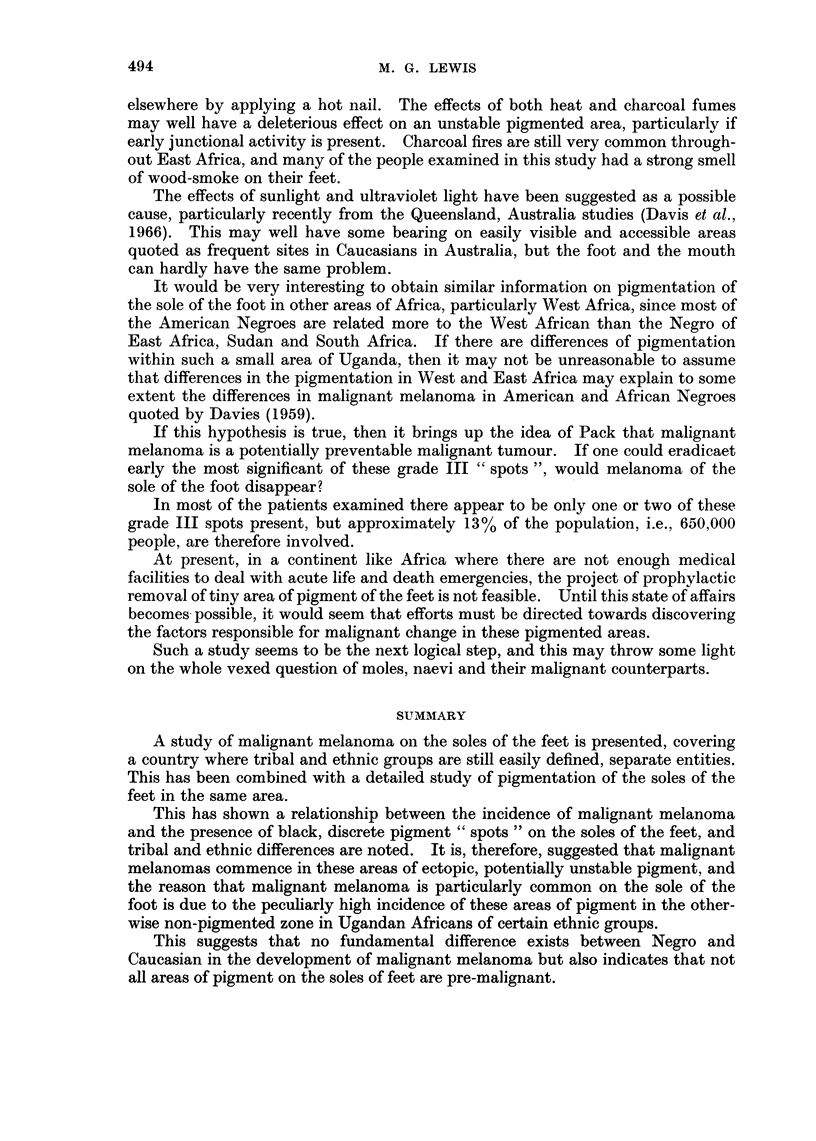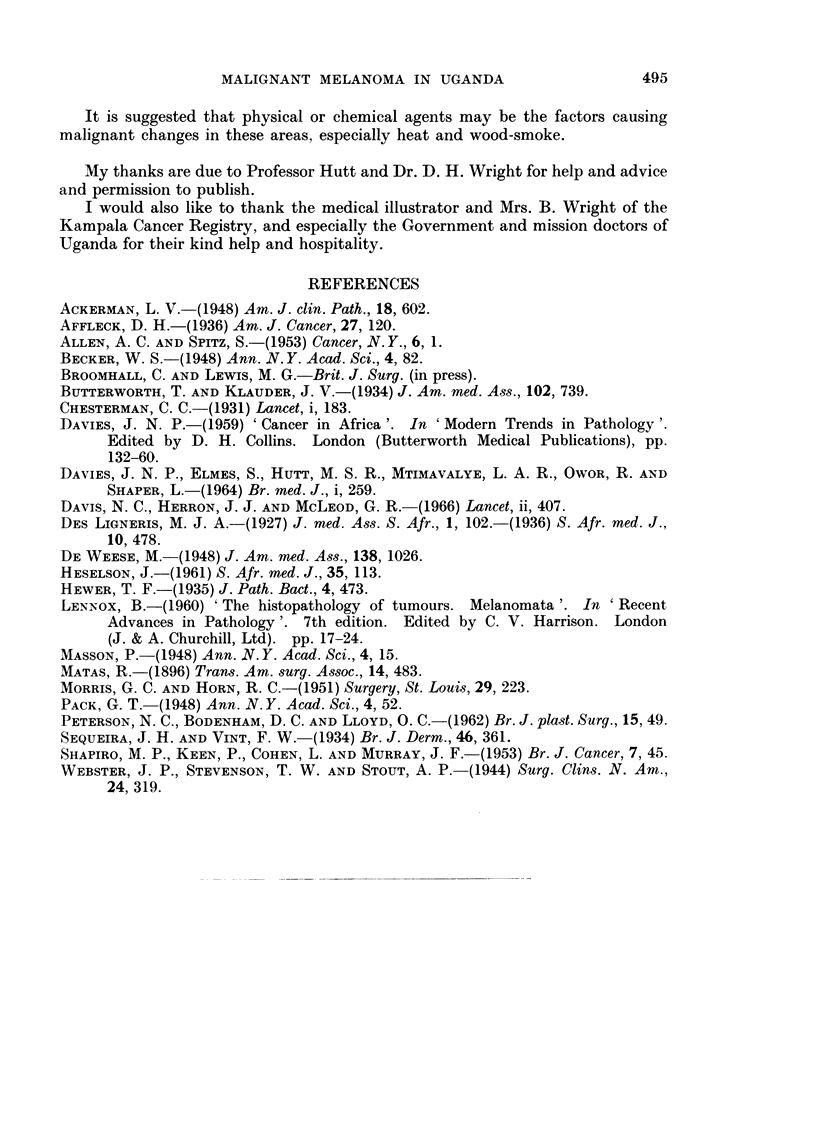# Malignant melanoma in Uganda. (The relationship between pigmentation and malignant melanoma on the soles of the feet).

**DOI:** 10.1038/bjc.1967.56

**Published:** 1967-09

**Authors:** M. G. Lewis

## Abstract

**Images:**


### MALIGNANT MELANOMA IN UGANDA

### (THE RELATIONSHIP BETWEEN PIGMENTATION AND MALIGNANT MELANOMA ON THE SOLES OF THE FEET)

M. G. LEWIS

*From the Department of Pathology, Medical School, Makerere University College, Kampala, Uganda*

Received for publication February 8, 1967

ONE of the earliest recorded cases of malignant melanoma in Africa is found in the Mengo Hospital notes of Sir Albert Cook in 1904 (Davies *et al.*, 1964). The writings of Sequeira and Vint in Kenya (1934), Des Ligneris in South Africa (1927) and Hewer in the Sudan (1935) showed that this tumour was not the rarity in African Negroes that earlier work on American Negro incidence had suggested (Matas, 1896). The common predilection for the sole of the foot was also emphasised by these writers, and Hewer made the suggestion that trauma, mainly in the form of thorn pricks, might be the cause.

The differences in the incidence in American and African Negroes was considered to be reflected in the differences in trauma to the sole of the foot and the wearing of shoes.

On the other hand, information from South Africa indicates that the incidence of malignant melanoma in Bantu and Caucasians is very similar (Shapiro, 1953). Davies quotes that the incidence of melanoma in South African Negroes in the Johannesburg area has not decreased with urbanisation and shoe wearing, but squamous carcinoma has decreased (Davies, 1959).

### *Moles and pigmentation*

The problem of moles, naevi and melanoma has long been controversial. Indeed, the very origins of these curious pigmented lesions have been the cause of dispute.

Masson (1948) postulated the neural crest hypothesis, and others, such as Unna and Bloch, believed naeval cells to be derived within the epidermis (Becker, 1948).

Whatever their origin, most people are agreed that moles and malignant melanoma are related, though the number of malignant melanomas thought to be derived from pre-existing moles varies in different surveys.

Allen and Spitz (1953) state that with a few exceptions such as malignant blue naevi, all melanocarcinomas of skin and mucous membranes arise in junctional or compound naevi. Ackerman (1948), Affleck (1936) and Webster *et al.* (1944), and Lennox (1960) state that less than 50% of malignant melanomas arise in previously existing naevi, and Becker (1948) records less than 25%.

Regarding the relationship of malignant melanoma of the sole of the foot and the presence of naevi, even more controversy exists.

Allen and Spitz maintain that for all practical purposes, pigmented blemishes of the genitalia, soles of the feet and palms should be considered to be wholly or at least in part junctional. Hewer states that in Sudanese Negroes the presence of malignant melanoma of the sole of the foot is probably due to trauma and dismisses the likelihood that pigmentation is in any way involved. He goes on to state that 95% of Sudanese Africans taken at random have easily detectable pigmented naevi, but does not state the sites and does not mention pigmented areas on the feet.

Pack (1948) makes the statement that naevi are uncommon in the pigmented races and that the Negro is particularly free from error in the distribution of pigmented cells or neoplastic groups of such cells.

Butterworth and Klauder (1934) showed that when one considers the number of malignant melanomas known to have arisen in a previous naevus the following results are obtained:

| | |
|---|---|
| Head | 16·5% |
| Neck | 7·7% |
| Trunk | 15·5% |
| Genitals | 2·7% |
| Foot | 52·3% |

When one considers that the large series of Allen and Spitz showed an incidence of 10·2% malignant melanoma on the soles of the feet, then the relative danger of pigmentation on the soles is even more striking. Heselson (1961) in South Africa showed the following results of a survey of moles and melanomas:

| Malignant melanoma | | Moles |
|---|---|---|
| 2 | to | 1 (Head and neck) |
| 30 | to | 1 (Genitalia) |
| 50 | to | 1 (Sole foot) |

In view of some of these controversies and impressions, it was decided to study the soles of the feet in Ugandan Africans and to try and relate the presence of pigmentation to malignant melanoma similarly to that described for malignant melanoma of the oral cavity (Broomhall and Lewis, in press).

A pilot study of 100 patients in Mulago Hospital showed a surprisingly high incidence of discrete areas of pigment on the soles of the feet. It was, therefore, decided to investigate the feet of representative tribes in Uganda and to relate them to the biopsy incidence of malignant melanoma in the same site.

### MATERIALS AND METHODS

Slides and records from the Kampala Cancer Registry and the biopsy records of Makerere Medical College, Pathology Department, together with personally received information and biopsies from Mulago Hospital and all the hospitals in Uganda furnish the pathological material on which this study is based. The Pathology Department of Makarere Medical College now receives all the biopsy material for the whole of Uganda and adjacent areas of neighbouring countries, so that a picture of malignant melanoma in a complete country is possible. In addition to this, a survey of pigmentation of the soles of the feet was carried out in most of the hospitals of Uganda. One hundred patients taken at random were examined in each hospital. Children less than ten years of age were excluded from this aspect of the study.

The types and degrees of pigmentation are recorded, with age, sex, tribe and district. The classification adopted is as follows:

Grade I = No pigment seen (Fig. 1).
Grade II = Areas of light brown to dark brown pigment of various sizes, often with irregular outlines (Fig. 2, 3).
Grade III = Discrete, small black areas of pigment with clear-cut margins (Fig. 4, 5).

### RESULTS AND OBSERVATIONS

The analysis of site and sex is summarised in Table I. The age incidence is shown in Fig. 6. Fig. 7 shows the tribal areas of Uganda under discussion with the distribution of melanomas of the feet throughout Uganda in the period from 1963–1966. Tribes from which the biopsies were sent are indicated, not the actual place of origin of the patient, which is often difficult to locate with any accuracy.

TABLE I.—*Distribution by Site and Sex of Malignant Melanoma in Uganda 1963–66 (Biopsy Cases)*

| Site | Male | Female | Total |
|---|---|---|---|
| Foot | 47 (48%) | 50 (52%) | 97 (64%) |
| Leg | 7 | 12 | 19* |
| Arm | 1 | 2 | 3 |
| Hand | 3 | 1 | 4 |
| Mouth | 3 | 4 | 7 |
| Nose | 3 | 1 | 4 |
| Eyelid | 1 | 0 | 1 |
| Genitals | 1 | 3 | 4 |
| Skin (other) | 7 | 6 | 13 |
| Totals | 73 (48%) | 79 (52%) | 152 |

* Seven of these presented as secondaries in the groin.

### *Site of melanomas*

Table I shows the analysis of the cases of malignant melanoma recorded in the period 1963–66. Again, the high incidence of melanoma of the sole of the foot can be seen. The figure of 64% is a conservative estimate, since the site was not always recorded, and several of those designated as " leg " may well have arisen on the foot. The same is true for the seven cases where the primary site was recorded as " groin ". Reviewing the slides of these cases showed lymph node metastases. Illustrative case 1 shows another example of a small primary malignant melanoma on the foot presenting in this way, and two others have similarly been identified in the past year.

The thirteen cases stated as " skin (other) " include examples where the site was only recorded as " skin ".

The sites which therefore stand out in this study are feet and mouth.

### *Age and sex*

The age distribution does not show any particular differences from previous series in both Africans and Caucasians. The sexes are almost equal.

*Tribal distribution of melanoma of feet*

The tribes of Uganda are derived from several ethnic groups, including Bantu, Hamitic, Nilotic and Nilo-Hamitic.

FIG. 6.—Age distribution of malignant melanoma in Uganda (1963–66).

The depth of pigmentation of the skin varies greatly in different groups, but the pigmentation, particularly grade III " spots " varies independently of this skin pigmentation.

The incidence figure in terms of population, particularly keeping in mind the number of cases that may never come to biopsy in underdeveloped areas, is comparable to that quoted by Peterson, Bodenham and Lloyd (1962) for the

---

EXPLANATION OF PLATES

FIG. 1.—Grade I. Sole of foot. Nil pigment.
FIG. 2.—Grade II. Pigmentation showing patches of light pigment varying in size and shape.
FIG. 3.—Grade II. Pigmentation. Patchy pigment of various shades of brown.
FIG. 4, 5.—Grade III. Pigmentation. Black, discrete, small, well-circumscribed pigmentation.
FIG. 12.—Small primary malignant melanoma on heel. Note melanotic " blush " and the grade III lesion adjacent.
FIG. 13.—Malignant melanoma of ball of foot (by kind permission of Dr. E. Williams, Kuluva).
FIG. 14.—Malignant melanoma of heel, close to junction of pigmented and non-pigmented skin.
FIG. 15.—Large, fungating, pigmented tumour arising from plantar aspect of second toe.
FIG. 16.—Transection of tumour from previous case showing intense black pigment.
FIG. 17.—Photomicrograph of grade III spot showing changes of junctional activity.

1 3 2

Lewis.

4

5

12

Lewis.

13

14

15

16

17

South-western region of England, where the incidence is 1·7/100,000; the Ugandan average between 1·4/100,000, with regional averages up to 3·8/100,000. The sex ratio is almost equal in 52% female, 48% male.

The results of the pigmentation survey of African feet are shown in Fig. 8, together with the incidence of malignant melanoma of the same site in each tribal area (a total of about 2000 pairs of feet).

FIG. 7.—Map showing tribal distribution of malignant melanoma of foot in Uganda (1963–66).

The total number of grade III lesions (258) are also presented diagrammatically in Fig. 9 to show the scatter of these lesions on the foot in general. In addition to this, Fig. 10 and 11 show the distribution of these lesions in two different Ugandan tribes, together with the sites of as many malignant melanomas available in these tribes where the site was accurately known.

A smaller survey of pigmentation of the hands is included to see if a similar relationship between melanomas in this site and pigmented " spots " occurs. One hundred adult patients taken at random in Mulago Hospital of all tribes gave a result:

Grade I — 54%
Grade II — 46%
Grade III — 10%

If the fingers alone are considered, 6% of patients have grade III spots.

The total number of melanomas of the hand seen during this period (Table I) is 4, i.e., 2·6% of all melanomas.

Although these numbers are small, there appears to be a 20 fold difference in malignant melanoma of the hands and feet, but a 4 fold difference in grade III

FIG. 8.—Incidence of malignant melanoma of the foot (upper part of figure) related to percentage incidence of the various grades of pigmentation in the tribes studied (lower part of figure).

pigment spots. This suggests that grade III lesions of the feet are potentially more likely to develop into malignant melanoma than similar areas on the hands.

The results of the pigmentation survey of feet from birth to the age of 20 is presented to attempt to show the natural history and development of these various types of pigmentation.

This shows a steady rise of grade II pigmentation from birth to 15 years of age and then remains at the average adult levels. Grade III pigmentation, however, does not appear in any appreciable extent until about 18–20 years of age. These results bring more support to the contention that grades II and III are different and unrelated. The appearance of grade III lesions after puberty is characteristic of naevi.

It is concluded, therefore, that the grade II pigment is a simple melanosis, and histology confirms this impression. There is no association between the melanosis and junctional activity or malignant melanoma.

FIG. 9.—Distribution of total number of grade III lesions.

*Illustrative Cases*

These brief descriptions are included to show the features of some of the lesions described in the text and to illustrate some of the changes in the adjacent skin, with particular reference to the pigmented areas described on the sole of the foot.

*Case* 1

A 60 year old female Mugishu was admitted to Mbale Hospital with a two-month history of swelling in the left groin. On examination an irregular mass of tumour was obvious and visible. In addition to this the patient had a solitary nodule palpable in the liver, just below the ninth costal cartilage. Biopsy of the mass in the groin revealed lymph nodes largely replaced by malignant melanoma, with marked amounts of melanin, both free in the macrophages and in tumour cells; there were no junctional changes in the overlying skin. The primary lesions were then revealed on close examination of the sole of the left foot (Fig. 12), where several discrete grade III pigmented lesions can be seen, and a small malignant melanoma in the centre of the sole of the heel surrounded by a " melanotic blush ".

FIG. 10.—Distribution of grade III lesions in two Ugandan tribes.

It is certainly unusual to find such an apparently early lesion on the sole, particularly with such evidence of widespread metastases.

This case illustrates how a melanoma could well have arisen from the grade III pigmented spots.

*Case* 2

A 35 year old male Lugbara was admitted to Kuluva Hospital with a fungating tumour on the ball of the left foot (Fig. 13) (note the pigment spots in the area away from the tumour), which he stated had been present for one month.

Biopsy confirmed the diagnosis of malignant melanoma.

In this case, the size of the tumour makes deduction about its origin difficult, but the position in relationship to the incidence of grade III lesions in this tribe and the evidence of grade III lesions in this patient is interesting (Fig. 10, 11).

FIG. 11.—Distribution of some malignant melanomas in the two Ugandan tribes shown in Fig. 10.

*Case* 3

A 30 year old female Muganda was admitted to Mulago Hospital with a history of 3 years' previously having noticed a small black area which subsequently ulcerated and slowly enlarged. On examination a large, circular, pigmented tumour was seen on the right heel (Fig. 14) near the junction of pigmented and non-pigmented skin.

Excision of the tumour, with skin grafting and block dissection of right groin glands was performed.

Histopathology showed a typical malignant melanoma. The regional lymph nodes contained no metastatic growth.

*Case* 4

A 50 year old male Muganda was admitted to Mulago Hospital with a history of growth on the right third toe.

On examination there was a large, irregular, fundating tumour 10 × 8 × 6 cm. (Fig. 15). Amputation was performed, and Fig. 16 shows a transection of the specimen, showing the intense blackness of the lesions.

Histopathology showed a typical heavily pigmented malignant melanoma.

This case shows the advanced stage at which these tumours are often seen, yet despite this, the paucity of obvious metastatic growth on presentation, in contrast to case 1.

### DISCUSSION

These observations strongly suggest that malignant melanoma on the sole of the foot in Ugandan Africans is closely related to the particularly high incidence of pigmented areas on the sole, and it is very likely that most of these melanomas arise in such areas. This would explain the particularly high incidence of this tumour on the sole of the foot. In support of this contention are the findings of Butterworth and Klauder (1934) and Allen and Spitz (1953), both of whom stated that there was a close relationship between pigmented moles and the danger of malignancy, particularly on the sole of the foot in Caucasians.

Hewer (1935), on the other hand, believed that trauma was the most likely cause and dismissed the likelihood of pigmentation playing any great part.

Although it is impossible to make direct comparisons between different hospitals in Uganda, since the size of population served by hospital and the local custom of the people vary from area to area, personal contact with up-country surgeons convinced me that there are differences in the incidence of melanoma, particularly on the foot, and that differences in tribal customs and individual doctors' biopsy rates did not play a large part in their differences. The population structure of the various tribal areas obviously must influence the rate of any neoplasm, but a glance at the map and use of the 1959 census figures suggests that there are still differences not explained on the basis of population.

The results of the pigmentation survey seem to be the best explanation, and Fig. 8 shows that as the incidence, particularly of grade III lesions, rises, the malignant melanoma rate also rises.

The question then appears to be, " Do these grade III pigmented areas reflect the anatomical site incidence of malignant melanoma on the foot? "

All the grade III lesions recorded in this survey have been summarised in position on Fig. 9.

Even with these small numbers, it can be seen that the common sites (particularly along the junction of pigmented dorsum and non-pigmented sole, and on heel, toes and ball of great toe) are common to both malignant melanoma and grade III pigmentation. These findings confirm the clinical impression that these sites on the feet are common for malignant melanoma (Burkitt, personal communication; Davies, 1959).

An interesting observation becomes apparent when the grade III lesions for individual tribal areas are plotted. Fig. 10 shows the incidence of such lesions from Kuluva Hospital, West Nile, in the Lugbara tribe. The pigment is mostly scattered in the anterior part of the sole of the foot, particularly on the ball of the great toe and the lateral aspect of the foot. The malignant melanomas from the same tribal area (Fig. 11) also show a tendency to occur at these sites (see illustrative case 3). In contrast to this are grade III lesions in the Baganda (Fig. 10), which when compared with the melanoma, show a comparable distribution on the heel.

The next question that arises is the nature of these areas of pigmentation. Are they moles (naevi), and is there junctional activity?

It is obviously impossible to obtain many biopsies from living patients, so that a survey is being undertaken in the autopsy room of Mulago Hospital. The following is in the form of a preliminary communication, for up to the present only fifty have been biopsied. These studies confirm the presence of melanin in the naked eye description of grades II and III. In fact every case designated as grade II or grade III has so far shown melanin in the basal epidermis.

In the relatively few grade III lesions studied so far in histological detail, changes varying from increased numbers of melanocytes to " clear cell " hyperplasia and actual junctional activity have been noted. In one case (Fig. 17), the appearance is consistent with a junctional naevus.

I have so far avoided calling these pigmented blemishes by any of the conventional terms, such as mole, naevus or lentigo, since there seem to be controversies over their usage and interpretation. I will, therefore, refer to these pigmented areas, grade I, II or III, as described.

Even before histological examination of the various grades of pigment is studied in detail, it can be seen from the evidence in Fig. 8 that only the grade III lesions are in any way statistically related to malignant melanoma, for the grade II pigment varies independently. The grade I (nil pigment) also shows no consistent relationship, so that it would seem that the mere absence of pigmentation is not the cause of melanomas developing on the sole of the foot. There also appears to be no relationship in terms of age or appearance between grade II and grade III lesions. In fact grade III lesions often appeared on otherwise non-pigmented or slightly pigmented soles. Therefore, not all pigment on the sole of the foot is potentially malignant, only the grade III areas.

I would, therefore, like to postulate that the fundamental reason why malignant melanoma is common, particularly in the African, on the soles of the feet, is the high incidence of potentially unstable collections of melanocytes seen in this area, and that this is genetically determined.

The problem of what makes these areas of pigmentation develop junctional and then malignant change is yet to be fully determined. Trauma may well play a part, and often patients tell of thorn pricks and such like. On the other hand, the wearing of shoes in urbanised Africans does not seem to have altered the incidence of melanoma on the foot, and the relative frequency even on Caucasian feet reported by Allen and Spitz (1953) and Pack (1948) and others does not support this theory. This study also shows no positive correlation between shoe-wearing and melanomas.

Chesterman (1931) suggested the relationship between crab-yaws and melanomas on the heel on the strength of three cases. Although I encountered crab-yaws on a few occasions in Uganda, there was no relationship between the disease and melanomas of pigmentation. In his paper, however, Chesterman does mention that at least two of the patients noted " black spots ", and these were " treated " by holding the foot over a smoking fire and the application of hot irons.

It is also a custom throughout parts of Uganda to treat lesions of the feet and elsewhere by applying a hot nail. The effects of both heat and charcoal fumes may well have a deleterious effect on an unstable pigmented area, particularly if early junctional activity is present. Charcoal fires are still very common throughout East Africa, and many of the people examined in this study had a strong smell of wood-smoke on their feet.

The effects of sunlight and ultraviolet light have been suggested as a possible cause, particularly recently from the Queensland, Australia studies (Davis *et al.*, 1966). This may well have some bearing on easily visible and accessible areas quoted as frequent sites in Caucasians in Australia, but the foot and the mouth can hardly have the same problem.

It would be very interesting to obtain similar information on pigmentation of the sole of the foot in other areas of Africa, particularly West Africa, since most of the American Negroes are related more to the West African than the Negro of East Africa, Sudan and South Africa. If there are differences of pigmentation within such a small area of Uganda, then it may not be unreasonable to assume that differences in the pigmentation in West and East Africa may explain to some extent the differences in malignant melanoma in American and African Negroes quoted by Davies (1959).

If this hypothesis is true, then it brings up the idea of Pack that malignant melanoma is a potentially preventable malignant tumour. If one could eradicaet early the most significant of these grade III " spots ", would melanoma of the sole of the foot disappear?

In most of the patients examined there appear to be only one or two of these grade III spots present, but approximately 13% of the population, i.e., 650,000 people, are therefore involved.

At present, in a continent like Africa where there are not enough medical facilities to deal with acute life and death emergencies, the project of prophylactic removal of tiny area of pigment of the feet is not feasible. Until this state of affairs becomes possible, it would seem that efforts must be directed towards discovering the factors responsible for malignant change in these pigmented areas.

Such a study seems to be the next logical step, and this may throw some light on the whole vexed question of moles, naevi and their malignant counterparts.

### SUMMARY

A study of malignant melanoma on the soles of the feet is presented, covering a country where tribal and ethnic groups are still easily defined, separate entities. This has been combined with a detailed study of pigmentation of the soles of the feet in the same area.

This has shown a relationship between the incidence of malignant melanoma and the presence of black, discrete pigment " spots " on the soles of the feet, and tribal and ethnic differences are noted. It is, therefore, suggested that malignant melanomas commence in these areas of ectopic, potentially unstable pigment, and the reason that malignant melanoma is particularly common on the sole of the foot is due to the peculiarly high incidence of these areas of pigment in the otherwise non-pigmented zone in Ugandan Africans of certain ethnic groups.

This suggests that no fundamental difference exists between Negro and Caucasian in the development of malignant melanoma but also indicates that not all areas of pigment on the soles of feet are pre-malignant.

It is suggested that physical or chemical agents may be the factors causing malignant changes in these areas, especially heat and wood-smoke.

My thanks are due to Professor Hutt and Dr. D. H. Wright for help and advice and permission to publish.

I would also like to thank the medical illustrator and Mrs. B. Wright of the Kampala Cancer Registry, and especially the Government and mission doctors of Uganda for their kind help and hospitality.

### REFERENCES

ACKERMAN, L. V.—(1948) *Am. J. clin. Path.*, **18**, 602.
AFFLECK, D. H.—(1936) *Am. J. Cancer*, **27**, 120.
ALLEN, A. C. AND SPITZ, S.—(1953) *Cancer, N.Y.*, **6**, 1.
BECKER, W. S.—(1948) *Ann. N.Y. Acad. Sci.*, **4**, 82.
BROOMHALL, C. AND LEWIS, M. G.—*Brit. J. Surg.* (in press).
BUTTERWORTH, T. AND KLAUDER, J. V.—(1934) *J. Am. med. Ass.*, **102**, 739.
CHESTERMAN, C. C.—(1931) *Lancet*, i, 183.
DAVIES, J. N. P.—(1959) 'Cancer in Africa'. *In* 'Modern Trends in Pathology'. Edited by D. H. Collins. London (Butterworth Medical Publications), pp. 132–60.
DAVIES, J. N. P., ELMES, S., HUTT, M. S. R., MTIMAVALYE, L. A. R., OWOR, R. AND SHAPER, L.—(1964) *Br. med. J.*, i, 259.
DAVIS, N. C., HERRON, J. J. AND MCLEOD, G. R.—(1966) *Lancet*, ii, 407.
DES LIGNERIS, M. J. A.—(1927) *J. med. Ass. S. Afr.*, **1**, 102.—(1936) *S. Afr. med. J.*, **10**, 478.
DE WEESE, M.—(1948) *J. Am. med. Ass.*, **138**, 1026.
HESELSON, J.—(1961) *S. Afr. med. J.*, **35**, 113.
HEWER, T. F.—(1935) *J. Path. Bact.*, **4**, 473.
LENNOX, B.—(1960) 'The histopathology of tumours. Melanomata'. *In* 'Recent Advances in Pathology'. 7th edition. Edited by C. V. Harrison. London (J. & A. Churchill, Ltd). pp. 17–24.
MASSON, P.—(1948) *Ann. N.Y. Acad. Sci.*, **4**, 15.
MATAS, R.—(1896) *Trans. Am. surg. Assoc.*, **14**, 483.
MORRIS, G. C. AND HORN, R. C.—(1951) *Surgery, St. Louis*, **29**, 223.
PACK, G. T.—(1948) *Ann. N.Y. Acad. Sci.*, **4**, 52.
PETERSON, N. C., BODENHAM, D. C. AND LLOYD, O. C.—(1962) *Br. J. plast. Surg.*, **15**, 49.
SEQUEIRA, J. H. AND VINT, F. W.—(1934) *Br. J. Derm.*, **46**, 361.
SHAPIRO, M. P., KEEN, P., COHEN, L. AND MURRAY, J. F.—(1953) *Br. J. Cancer*, **7**, 45.
WEBSTER, J. P., STEVENSON, T. W. AND STOUT, A. P.—(1944) *Surg. Clins. N. Am.*, **24**, 319.